# Neurocardiac regulation: from cardiac mechanisms to novel therapeutic approaches

**DOI:** 10.1113/JP276962

**Published:** 2018-11-12

**Authors:** E. N. Bardsley, D. J. Paterson

**Affiliations:** ^1^ Wellcome Trust OXION Initiative in Ion Channels and Disease Oxford UK; ^2^ Burdon Sanderson Cardiac Science Centre, Department of Physiology Anatomy and Genetics, University of Oxford Oxford OX1 3PT UK

**Keywords:** Autonomic Nervous System, Sympathetic Ganglion, Hypertension, Cardiovascular Disease, Cyclic Nucleotide, Protein Kinase, Sympathetic Nervous System, Intracellular Calcium

## Abstract

Cardiac sympathetic overactivity is a well‐established contributor to the progression of neurogenic hypertension and heart failure, yet the underlying pathophysiology remains unclear. Recent studies have highlighted the importance of acutely regulated cyclic nucleotides and their effectors in the control of intracellular calcium and exocytosis. Emerging evidence now suggests that a significant component of sympathetic overactivity and enhanced transmission may arise from impaired cyclic nucleotide signalling, resulting from compromised phosphodiesterase activity, as well as alterations in receptor‐coupled G‐protein activation. In this review, we address some of the key cellular and molecular pathways that contribute to sympathetic overactivity in hypertension and discuss their potential for therapeutic targeting.

## Graphical abstract

In healthy stellate neurons, Ca^2+^‐dependent exocytosis facilitates the release of noradrenaline (NA) onto cardiac myocytes, where post‐synaptic β_1_‐ARs and β_2_‐ARs are activated. Increases in extracellular NA acts on presynaptic α_2_‐ARs, and presynaptic β_1_‐ARs and β_2_‐ARs to a small degree. Acute regulation of cAMP and cGMP signalling is maintained by phosphodiesterases (PDEs). cAMP generation and PKA activity increases [Ca^2+^]_i_ via phosphorylation of the N‐type Ca^2+^ Channel (I_*CaN*_; Ca_V_2.2); endoplasmic reticulum (ER) store and mitochondrial Ca^2+^ release. In diseased sympathetic neurons, Ca^2+^‐dependent exocytosis facilitates the release of NA and adrenaline (Adr) onto cardiac myocytes, where post‐synaptic β_2_‐AR signalling is upregulated and preferentially activated. Chronic elevations in NA and Adr release also acts on presynaptic β‐ARs. Adr preferentially stimulates the β_2_‐AR isoform, thus augmenting cAMP generation and PKA activity in prehypertension, in a potentiating feed‐forward manner. Increased PKA activity raises [Ca^2+^]_i_ via phosphorylation of the N‐type Ca^2+^ Channel (ICaN; Ca_V_2.2), exacerbating the Ca^2+^ phenotype that may contribute to the initiation of hypertension.

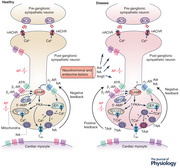

## Introduction

The autonomic nervous system, comprising the parasympathetic and sympathetic branches, provides a regulatory link between the central nervous system (CNS) and myocardium (Herring & Paterson, 2018). The notion of a mind–body connection has been proposed by many scientists throughout history, but it was perhaps first recorded in ad 30 by the Roman physician Celsus who wrote, ‘fear and anger and any other state of mind may often be apt to excite the pulse’ (Celsus & Spencer, 1935). Yet, the physiological mechanisms responsible for the relationship between the heart and the brain remained elusive until the 19th century, whereupon, it was discovered that heart rate could be accelerated or decelerated by stimulation of two antagonistic systems: sympathetic or parasympathetic nerve fibres (Gaskell, 1886; Langley, 1898; Woollard, 1926; Sheehan, 1936; Hoff, 1940). The ‘autonomic nervous system’, as coined by Langley in 1898 (Langley, 1898), is now known to play an integral role in cardiovascular homeostasis and cardiac responses to physical or emotional disturbances (Rozanski *et al*. 1999; Steptoe & Kivimaki, 2012; Tahsili‐Fahadan & Geocadin, 2017; Herring & Paterson, 2018).

The cervicothoracic sympathetic stellate ganglion located adjacently to T1–T4 preferentially innervates the heart (Gaskell, 1886; Korzina *et al*. 2011) and, as such, exerts the greatest control over heart rate acceleration, contractility and conduction velocity at the atrio‐ventricular node (Shivkumar *et al*. 2016). Chronic alteration in sympathetic/parasympathetic balance (dysautonomia) is a well‐established contributor to many cardiovascular diseases (CVDs) and is strongly linked to clinical outcome and prognosis (Brook & Julius, 2000; Palatini & Julius, 2004; Malpas, 2010; Parati & Esler, 2012; Mancia & Grassi, 2014). Increasing evidence suggests that essential hypertension is underpinned and maintained by sustained elevations in sympathetic nerve activity (SNA) and chronic end‐organ transmission (Iriuchijima, 1973; Judy *et al*. 1979; Esler *et al*. 1986; 1988; Grassi & Esler, 1999; Johansson *et al*. 1999; Guyenet, 2006; Wang *et al*. 2006; Malpas, 2010; Parati & Esler, 2012; Shanks *et al*. 2013 *b*; Esler, 2014; Oliveira‐Sales *et al*. 2014; Grassi *et al*. 2015; Oliveira‐Sales *et al*. 2016). Elevations in SNA are also frequently seen in normotensive progeny of hypertensive patients (Ferrara *et al*. 1988; Hausberg *et al*. 1998; Lopes *et al*. 2000; Piccirillo *et al*. 2000; Maver *et al*. 2004; Hamer, 2006; Pal *et al*. 2011; Johncy *et al*. 2015), suggesting a causative role and potential genetic basis (Judy *et al*. 1979; Horikoshi *et al*. 1985; Adams *et al*. 1989) for sympathetic overactivity in the aetiology of hypertension.

**Figure 1:**
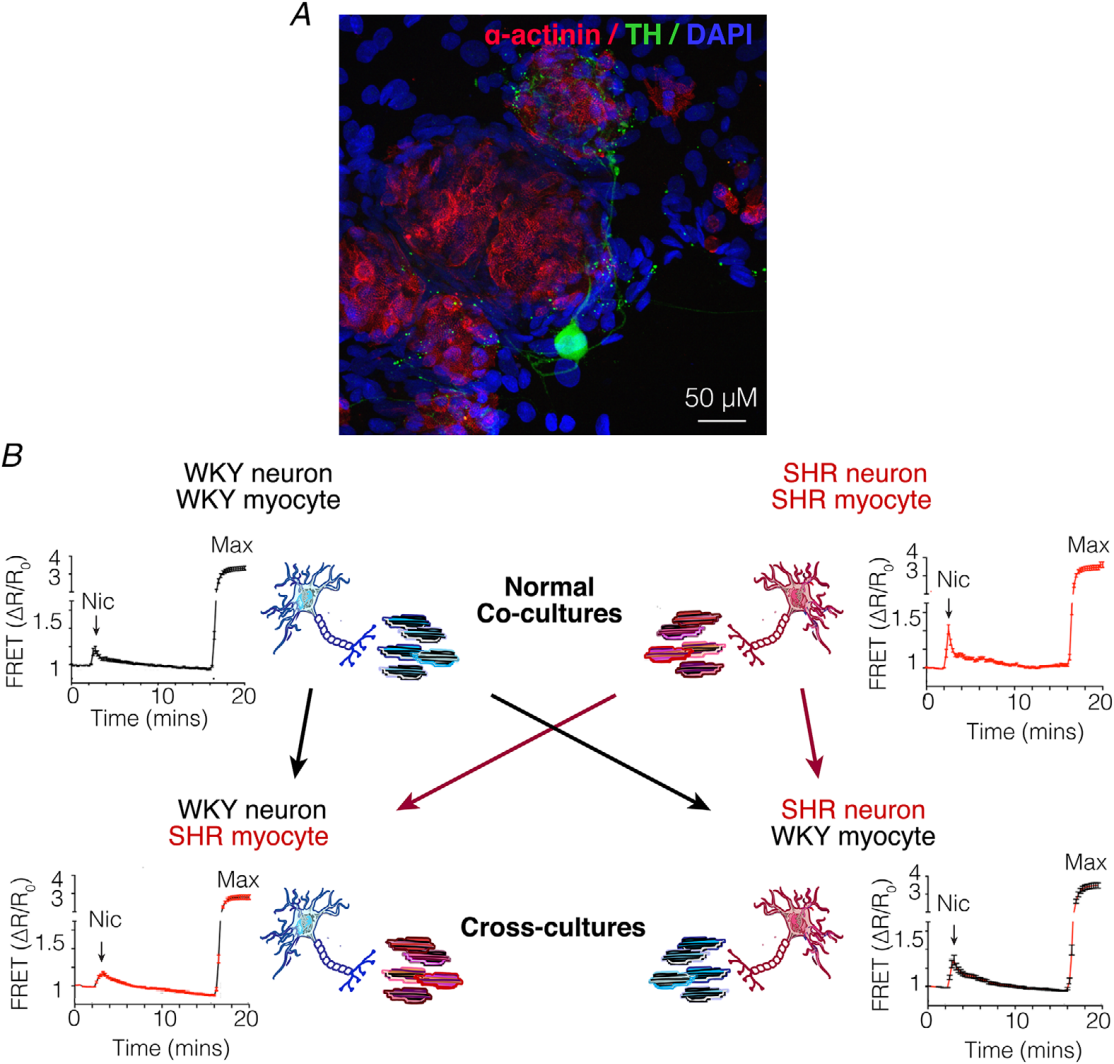
Sympathetic neurons are a powerful driver of myocyte function in cardiovascular disease *A*, immunofluorescence depicting a co‐culture of sympathetic neurons and ventricular myocytes (reproduced from Larsen *et al*. 2016 *b*). Sympathetic neurons labelled with tyrosine hydroxylase (TH, green) densely innervate cultured cardiomyocytes labelled with sarcomeric α‐actinin (red). *B*, Wistar–Kyoto (WKY) or SHR sympathetic neurons were stimulated with nicotine (Nic) and the resulting myocyte cAMP was measured as a surrogate for sympathetic transmission, in mycoytes transduced with a cAMP Förster resonance energy transfer (FRET) sensor. FRET sensors were maximally stimulated (max) with an adenylyl cyclase (AC) activator forskolin (25 M) and a non‐specific phosphodiesterase (PDE) inhibitor 3‐isobutyl‐1‐methylxanthine (IBMX,100 M). In healthy co‐cultures (WKYn/WKYm), neuron‐evoked myocyte cAMP (17.05 ± 3.715, *n* = 29 cells) was significantly lower than cAMP measured in the diseased co‐culture myocytes (SHRn/SHRm; 44.02 ± 5.310, *n* = 36 cells; *P* < 0.0001). Cross‐cultures were established by plating diseased SHR neurons on top of healthy WKY myocytes (SHRn/WKYm) or healthy WKY neurons on top of diseased SHR myocytes (WKYn/SHRm). In the first cross‐culture (SHRn/WKYm), neuronal stimulation elevated myocyte cAMP (31.37 ± 5.194, *n* = 42 cells) to levels that were not significantly different from measured in the diseased (SHRn/SHRm) co‐cultures (*P* = 0.094), demonstrating that enhanced neuronal transmission elevates healthy‐myocyte cAMP to levels observed in disease. Moreover, in the second cross‐culture (WKYn/SHRm), stimulation of WKY neurons elevated SHR myocyte cAMP (15.67 ± 1.936, *n* = 24 cells) to levels that were not significantly different from that measured in healthy (WKYn/WKYm) co‐cultures (*P* = 0.76), demonstrating that healthy neurons attenuate the elevated myocyte cAMP response observed in SHR myocytes (modified from Larsen *et al*. 2016 *b*).

However, it is also well established that SNA is not uniformly altered within each ganglionic site (Grassi *et al*. 2015) and preclinical models have highlighted the critical role of elevated cardiac sympathetic nerve activity, specifically in the initiation and maintenance of hypertension (Souza *et al*. 2001; Petersson *et al*. 2002; Tan *et al*. 2010; Shanks *et al*. 2013 *b*; Larsen *et al*. 2016 *a*; Tromp *et al*. 2018), cardiac arrhythmia (Meredith *et al*. 1991) and heart failure (Kaye *et al*. 1995; Rundqvist *et al*. 1997; Watson *et al*. 2007; Ramchandra *et al*. 2009; Tu *et al*. 2014). Multiple levels of the neural axis comprising several integrated feedback loops are involved in the regulation of autonomic transmission, and may be disturbed in hypertension. These include cardio‐cardiac reflexes and intrinsic cardiac nerve activity that alter end‐organ transmission within the myocardium directly, intrathoracic reflexes and feedback mechanisms that modify sympathetic ganglionic efferent transmission, and spinal and lower brainstem regulation that modulate autonomic outflow (Shivkumar *et al*. 2016; Hanna *et al*. 2017). Sustained alterations in one or several of these feedback processes may directly contribute to an elevation in SNA, yet it is difficult to dissociate the primary causative events from secondary consequential factors. Nevertheless, the dominance of cardiac sympathetic neurons over myocyte function is observed. This is illustrated in Fig. 1, where co‐cultures of diseased stellate neurons and myocytes from rats predisposed to hypertension display enhanced myocyte cyclic adenosine monophosphate (cAMP) generation during neuronal stimulation compared to normal co‐cultures (Larsen *et al*. 2016 *b*). Moreover, cross‐culturing diseased stellate neurons provokes healthy myocytes into a prehypertensive state partially recapitulating the elevation in cAMP observed in diseased myocytes. Critically, however, healthy neurons cultured with diseased myocytes rescues the aberrant myocardial cAMP response restoring cAMP to levels seen in normal myocytes (Larsen *et al*. 2016 *b*). What are the mechanisms that underpin the sympathetic phenotype and lead to elevated cardiac sympathetic transmission?

In models of neurogenic hypertension, several key sympathetic adaptations are reported, including increased neuronal firing rate and burst frequency (Iriuchijima, 1973; Briant *et al*. 2015), elevated and aberrant regulation of intracellular Ca^2+^ ([Ca^2+^]_i_) that facilitates exocytosis (Li *et al*. 2013; Larsen *et al*. 2016 *a*; Shanks *et al*. 2017; Tomek *et al*. 2017), decreased transmitter reuptake (Esler *et al*. 1981; Kimura *et al*. 1983; Esler *et al*. 1991; Rumantir *et al*. 2000 *b*; Shanks *et al*. 2013 *a*), and alterations in presynaptic feedback systems coupled to impaired intracellular signalling cascades (Wang *et al*. 2006; Shanks *et al*. 2013 *b*; Bardsley *et al*. 2018 *b*). In this brief review, we present the current evidence for the molecular and biochemical alterations that occur in stellate ganglia from rat and human patients that have a sympathetic phenotype and discuss their potential for therapeutic targeting.

### Intrinsic excitability: control by cyclic nucleotides

**Figure 2:**
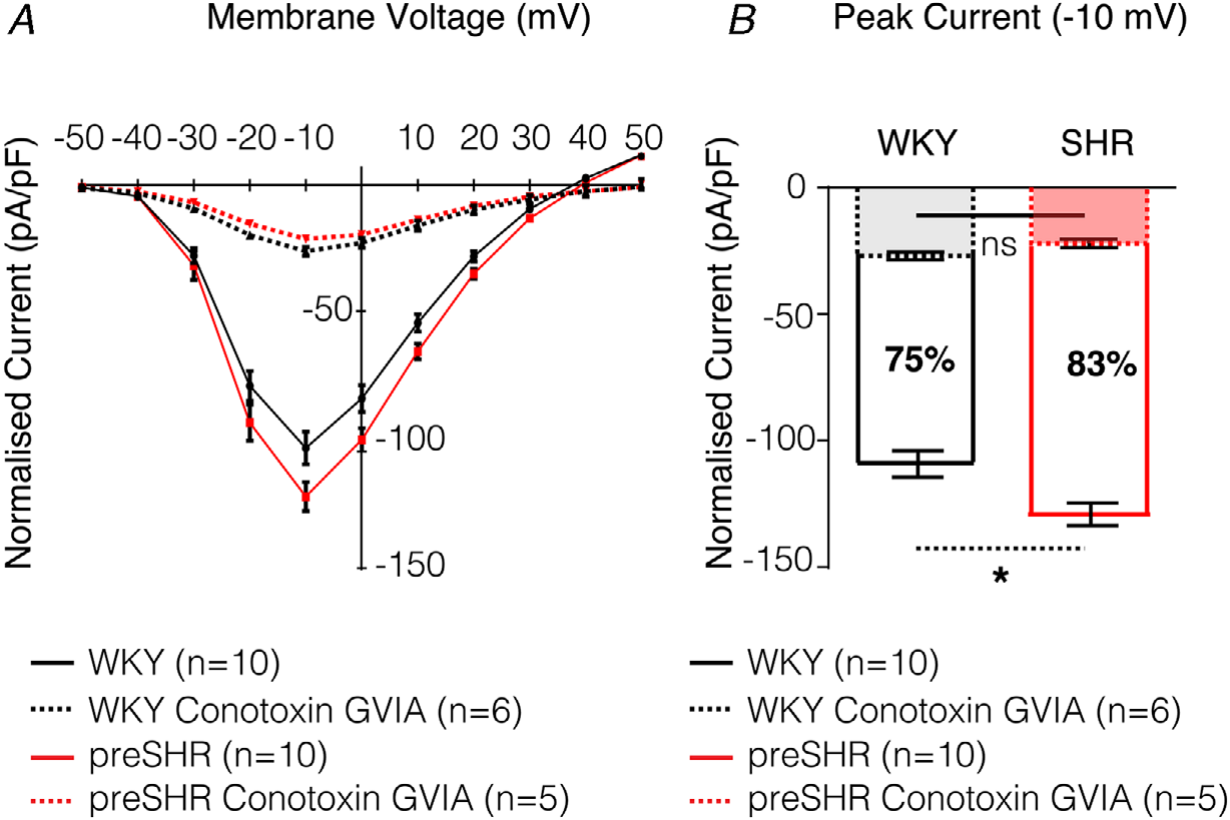
N‐type Ca^2+^ channel conductance is elevated in preSHR cardiac sympathetic neurons Whole cell voltage clamp was performed on cardiac sympathetic stellate neurons to investigate whole cell Ca^2+^ currents. *A*, the current–voltage relationship. Access to the cell was obtained in normal Tyrode's solution containing the following (in mM): 135 NaCl, 4.5 KCl, 11 glucose, 20 HEPES, 1MgCl_2_, 2 CaCl_2_, pH 7.4. To identify the Ca^2+^ current, normal Tyrode solution was replaced with a Ca^2+^‐isolating solution using Ba^2+^ as the charge carrier, containing the following (in mM): 135 TEACl, 10 HEPES, 4.5 KCl, 1 MgCl_2_, 4 glucose, 1 NaHCO_3_, 2 BaCl_2_, pH 7.40, either in the presence or absence of ω‐conotoxin GVIA (1 μM), which selectively blocks N‐Type Ca^2+^ channels (IC_50_ = 0.15 nM) (Sato *et al*. 1993). Ba^2+^ was used as the charge carrier to avoid Ca^2+^‐dependent current inactivation (Imredy & Yue, 1994). The internal solution contained the following (in mM): 140 CsCl, 10 HEPES, 0.1 CaCl_2_, 1 MgCl_2_, 4 MgATP, 1 EGTA, pH 7.30. All solutions had osmolarities of 300 mOsm L^−1^. *B*, the whole cell Ca^2+^ current is larger in preSHR sympathetic nerves (127.5 ± 5.94 pA pF^−1^, *n* = 10) compared to WKY cells (−108.0 ± 6.80 pA pF^−1^, *n* = 10, *P* = 0.045) where peak current was recorded at −10 mV. ω‐Conotoxin GVIA (1 μM), significantly reduced the N‐type Ca^2+^ channel current to similar levels in both strains. A 75% reduction was observed in cells cultured from WKY stellate ganglia (−26.88 ± 1.7 pA pF^−1^, *n* = 6) and an 83% reduction was measured in neurons cultured from preSHR ganglia (−22.04 ± 1.60 pA pF^−1^, *n* = 5, ns) where peak current remained at −10 mV. Solid lines represent the mean of the WKY (black) and preSHR (red) control data. Dashed lines represent the mean of WKY (black) and preSHR (red) in the presence of ω‐Conotoxin GVIA. Data are represented as mean ± SEM. (*A* and *B* modified from Larsen *et al*. 2016 *a*).

The N‐type Ca^2+^ channel is the primary neuronal voltage‐gated Ca^2+^ channel (Catterall, 2003, 2011) and as such plays a critical role in determining the cytosolic Ca^2+^ concentration during an action potential in sympathetic neurons (Pruneau & Bélichard, 1992; Ino *et al*. 2001; Mori *et al*. 2002; Uhrenholt & Nedergaard, 2003; Tu *et al*. 2014; Larsen *et al*. 2016 *a*). Emerging evidence suggests that N‐type Ca^2+^ channel activity is elevated in cardiac sympathetic ganglia in the prehypertensive SHR (Fig. 2; Larsen *et al*. 2016 *a*) and in heart failure (Tu *et al*. 2014), indicating a synaptopathy that augments intracellular Ca^2+^ and raises the intrinsic excitability of these nerves (Briant *et al*. 2015). Voltage‐gated Ca^2+^ channel conductance is differentially regulated by kinase phosphorylation (Gray *et al*. 1998; Schroder, 2003; Mahapatra *et al*. 2012; Larsen *et al*. 2016 *a*) where processes that decrease cyclic guanosine monophosphate (cGMP)–protein kinase G (PKG) signalling, or elevate cAMP–protein kinase A (PKA) signalling result in a net increase in Ca^2+^ channel conductance (Brown & Birnbaumer, 1988; Leiser & Fleischer, 1996; Gray *et al*. 1998; D'Ascenzo *et al*. 2002; Schroder, 2003; Mahapatra *et al*. 2012; Zamponi *et al*. 2015; Sandoval *et al*. 2017). Thus, processes that selectively modulate the strength of cAMP or cGMP signals effectively regulate neuronal transmission (Pruneau & Bélichard, 1992; Leiser & Fleischer, 1996; Gray *et al*. 1998; Molderings *et al*. 2000; Ino *et al*. 2001; Mori *et al*. 2002; Tanaka *et al*. 2013; Yamada *et al*. 2014).

### An increased cAMP–PKA/cGMP–PKG ratio exacerbates cardiac sympathetic activity

**Figure 3:**
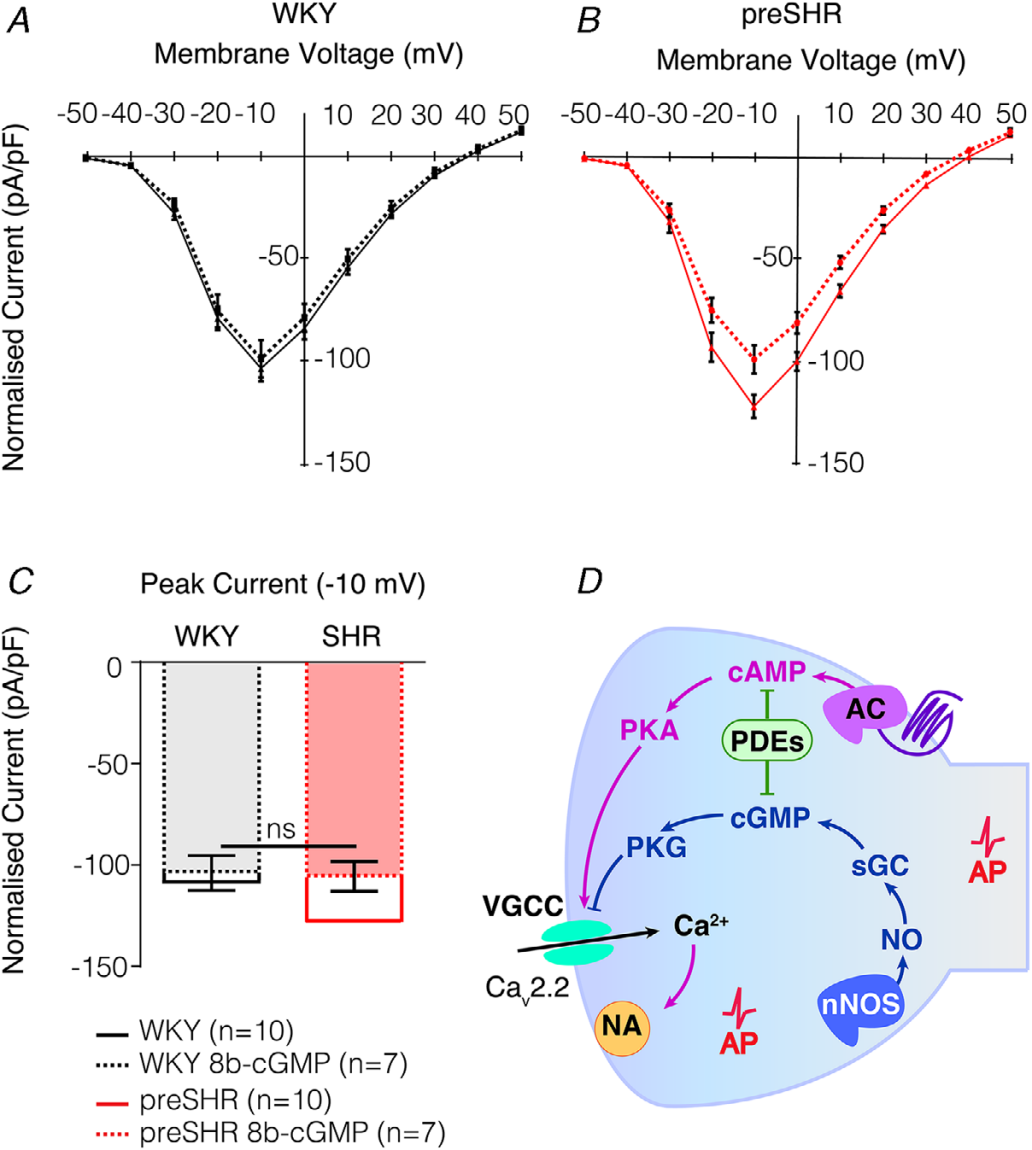
Elevated Ca^2+^ conductance in preSHR stellate neurons is rescued with cGMP administration *A*–*C*, to ascertain whether cGMP signalling inhibits Ca^2+^ currents, whole cell voltage clamp was performed on sympathetic neurons young normotensive WKY (*A*) and young prehypertensive SHR (*B*) in the presence of a cGMP analogue, 8‐bromo‐cGMP (8b‐cGMP) (Larsen *et al*. 2016 *a*). Access to the cell was obtained in normal Tyrode solution containing the following (in mM): 135 NaCl, 4.5 KCl, 11 glucose, 20 HEPES, 1 MgCl_2_, 2 CaCl_2_, pH 7.4. To identify the Ca^2+^ current, the solution was replaced with a Ca^2+^‐isolating solution using Ba^2+^ as the charge carrier, containing the following (in mM): 135 TEACl, 10 Hepes, 4.5 KCl, 1 MgCl_2_, 4 glucose, 1 NaHCO_3_, 2 BaCl_2_, pH 7.40, in either the presence or the absence of 8b‐cGMP (100 μM). Ba^2+^ was used as the charge carrier to avoid Ca^2+^‐dependent current inactivation (Imredy & Yue, 1994). The internal solution contained the following (in mM): 140 CsCl, 10 Hepes, 0.1 CaCl_2_, 1 MgCl_2_, 4 MgATP, 1 EGTA, pH 7.30. All solutions had osmolarities of 300 mOsm L^−1^. 8b‐cGMP significantly reduced the elevated preSHR Ca^2+^ currents (−127.5 ± 5.94 pA pF^−1^, *n* = 10 to −105.2 ± 7.79 pA pF^−1^, *n* = 7) to levels that were no longer greater than WKY Ca^2+^ currents (−108.0 ± 6.80 pA pF^−1^, *n* = 10). Moreover, 8b‐cGMP had no significant effect on the WKY Ca^2+^ current, where peak currents were measured at −10 mV. Continuous lines represent the mean of the WKY (black) and preSHR (red) control data. Dashed lines represent the mean of WKY (black) and preSHR (red) in the presence of 8b‐cGMP. Data are represented as mean ± SEM. (*A*‐*C* are reproduced from Larsen *et al*. 2016 *a*). *D*, model diagram representing N‐type Ca^2+^ channel control by PKA and PKG, where PKA augments and PKG inhibits channel conductance. Pathways that are decreased (blue) or increased (pink) in disease are represented. AP, action potential; NA, noradrenaline; VGCC, voltage‐gated calcium channel; AC, adenylyl cyclase.

Nitric oxide (NO) is a significant neuronal modulator of sympatho‐vagal activity (Sears *et al*. 1998; Wang *et al*. 2007). In the SHR, impaired NO generation via neuronal nitric oxide synthase (nNOS; Wang *et al*. 2007; Danson *et al*. 2009; Lee *et al*. 2009; Li *et al*. 2013, 2015; Lu *et al*. 2015) and down‐regulation of soluble guanylyl cyclase (sGC; Li *et al*. 2013; Bardsley *et al*. 2018 *a*) lead to significant reductions in cGMP production and PKG activity (Li *et al*. 2013, 2015; Larsen *et al*. 2016 *a*). In the prehypertensive rat, deficits in cGMP–PKG signalling are directly linked to elevations in N‐type Ca^2+^ channel Ca^2+^ conductance (Larsen *et al*. 2016 *a*; Fig. 3) and may contribute to the increased firing rate and spike amplitude observed in models of disease (Briant *et al*. 2014; Tu *et al*. 2014). To understand the genetic basis for these observations, we carried out a comprehensive RNA sequencing study using ganglia from hypertensive and normotensive rats (Bardsley *et al*. 2018 *a*) and found that transcripts within the cGMP–PKG pathway were significantly under‐represented in the stellate ganglia of SHR with established hypertension. Notable transcripts included down‐regulation of protein kinase G II (*Prkg2*) and the α1‐sGC subunit (*Gucy1a3*). Genome wide association studies (GWAS) have also revealed a critical link between mutations in loci containing the gene *Gucy1a3* and clinical hypertension (Ehret *et al*. 2011; Zheng *et al*. 2015; Wallace *et al*. 2016; Rippe *et al*. 2017; Seidel & Scholl, 2017), myocardial infarction (Erdmann *et al*. 2013; Wobst *et al*. 2015), atherosclerosis (Segura‐Puimedon *et al*. 2016; Wobst *et al*. 2016) and coronary artery disease (CARDIoGRAMplusC4D Consortium *et al*. 2013; Nikpay *et al*. 2015; Kessler *et al*. 2017).

Reductions in cGMP–PKG or increases in cAMP–PKA augment Ca^2+^ conductance (Fig. 3) via site‐specific phosphorylation of the N‐type Ca^2+^ channel, where a shift towards cAMP–PKA signalling in hypertension facilitates exocytosis (Leiser & Fleischer, 1996; Gray *et al*. 1998; D'Ascenzo *et al*. 2002; Tanaka *et al*. 2013; Larsen *et al*. 2016 *a*). In support of the evidence for elevated cAMP–PKA activity in hypertension, we identified a significant down‐regulation in the gene encoding the type Iα regulatory subunit of PKA (*Prkar1a*) in our RNA sequencing dataset. This subunit plays a dominant role as an endogenous inhibitor of kinase activity (Bardsley *et al*. 2018 *a*) where loss‐of‐function mutations in *Prkar1a* are associated with a twofold greater responsiveness to cAMP and an excess of PKA type II activity (Stratakis *et al*. 2001). Knock‐out mouse models of *Prkar1a* display impaired axonal sorting, myelination and proliferation (Guo *et al*. 2013). In humans, *Prkar1a* mutations are characterised by endocrine overactivity, neural dysfunction and cardiac complications, which result in dysregulation of arterial blood pressure homeostasis, arrhythmia and cardiomyopathies (Stratakis, 2002; Horvath *et al*. 2010), highlighting the importance of cAMP–PKA signalling in neuronal and cardiovascular regulation. Consequently, it appears that in cardiac sympathetic nerves from prehypertensive rats, several processes that favour excitatory cAMP–PKA signalling are up‐regulated, whereas pathways coupled to NO–cGMP are critically impaired early in disease, thus exacerbating or underpinning the observed Ca^2+^ phenotype (Li *et al*. 2013, 2015; Larsen *et al*. 2016 *a*; Fig. 3 *D*).

### Phosphodiesterase enzymes: the centre of balance for cyclic nucleotides

Phosphodiesterase enzymes (PDEs) regulate ion channel activity through selective termination of cAMP and/or cGMP signalling (Tanaka *et al*. 2013; Zhao *et al*. 2016); therefore, the acute spatial and temporal regulation of cyclic nucleotide (cN) levels by PDEs is critical for maintaining a fine balance between PKA‐ and/or PKG‐mediated effects (Zaccolo & Movsesian, 2007; Stangherlin & Zaccolo, 2012). The cN signal is acutely maintained by the PDE superfamily, comprising 11 isoforms (Stangherlin & Zaccolo, 2012), which confine individual and unique cAMP/cGMP signals to distinct subcellular compartments, enabling the regulation of multiple effector responses at any given time (Lefkimmiatis & Zaccolo, 2014). Indeed, cAMP is localised in close proximity to its effectors and regulators, where PKA, PDEs and phosphatases are tethered to A‐kinase anchoring proteins forming signalosomes that restrict the duration and magnitude of the cAMP–PKA signal within specific subcellular domains (Musheshe *et al*. 2018). Moreover, PDE isoforms are also subject to feedback inhibition and/or potentiation where specific isoforms are sensitive to cNs themselves (Zaccolo & Movsesian, 2007; Zhao *et al*. 2016), kinase activity (Zaccolo & Movsesian, 2007; Francis *et al*. 2011) and/or intracellular Ca^2+^/calmodulin‐dependent protein kinase signalling (Maurice, 2003; Bender, 2006; Francis *et al*. 2011). Sustained elevations in cAMP generation or alterations in PDE activity underpin several cardiovascular pathologies including cardiac hypertrophy (Zaccolo & Movsesian, 2007; Sprenger *et al*. 2015; Zoccarato *et al*. 2015) and sympathetic overactivity in hypertension (Larsen *et al*. 2016 *a*; Liu *et al*. 2018) where cAMP signals saturate the available PDEs and diffuse into neighbouring compartments leading to aberrant effector activity (Larsen *et al*. 2016 *a*; Zhao *et al*. 2017).

### Phosphodiesterases in the cardiac sympathetic ganglia

**Figure 4:**
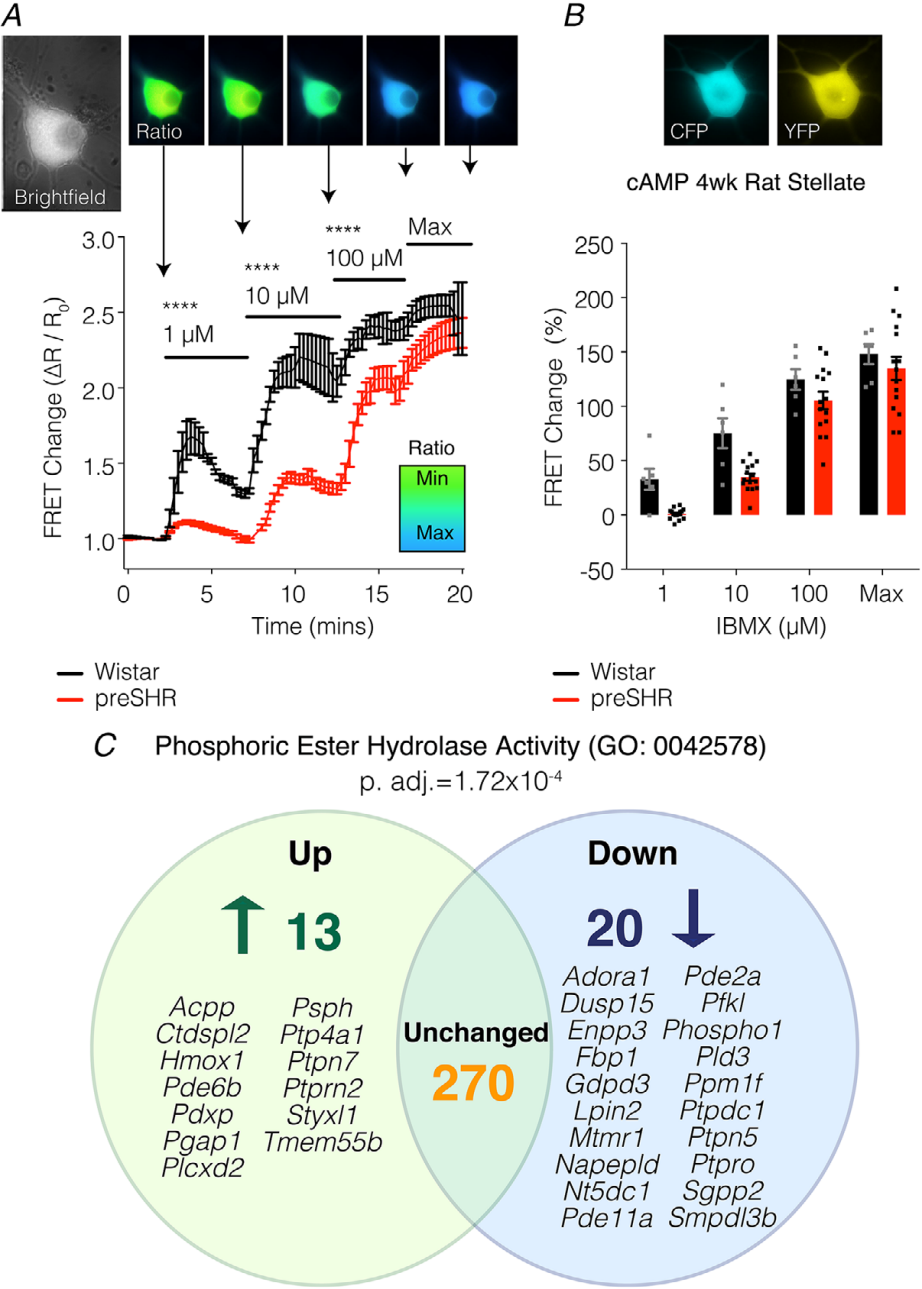
Phosphodiesterase (PDE) activity is impaired in preSHR neurons and has a genetic component *A* and *B*, to investigate whether cytosolic PDE signalling is impaired in preSHR sympathetic neurons, a non‐specific PDE inhibitor, 3‐isobutyl‐1‐methylxanthine (IBMX; inhibits PDEs 1–7, 10–11), was administered to sympathetic stellate neurons (1–100 μM). The resulting intracellular cAMP was measured using real‐time Förster resonance energy transfer (FRET) in cells transduced with the adenovirus encoding the Epac‐S^H187^ biosensor (Klarenbeek *et al*. 2015). *A*, there was significantly greater IBMX‐stimulated cAMP in Wistar *vs*. preSHR neurons at all concentrations measured (two‐way repeated measures ANOVA; *P* < 0.05) supporting the evidence that there is a differential PDE profile in preSHR *vs*. control stellate neurons. At 100 μM IBMX, FRET responses were close to sensor saturation. *B*, peak FRET changes are depicted. Data are expressed as mean ± SEM. *C*, we investigated whether transcriptomic changes could be identified in SHR stellate ganglia with established hypertension. Using RNA sequencing, it was observed that the molecular function gene ontology (GO) group encoding ‘phosphoric ester hydrolase activity’ (GO:0042578) was significantly over‐represented in the SHR ganglia at 16 weeks. Thirty‐three genes were found to be differentially expressed and many of these mapped to regulators of PDE and kinase activity (figure reproduced from Bardsley *et al*. 2018 *a*).

We have previously reported that the activity of specific PDEs involved in the cross‐talk between cAMP and cGMP pathways (PDE2a, PDE3) are impaired in cardiac sympathetic nerves in prehypertension (Li *et al*. 2015; Bardsley *et al*. 2016; Larsen *et al*. 2016 *a*), and that cGMP pathways are preferentially diminished (Larsen *et al*. 2016 *a*). However, a distinct contrast has also been identified in the hydrolysing activity of the wider PDE family within the sympathetic ganglia between normotensive and prehypertensive strains (Fig. 4 *A*). To understand the genetic basis for these observations, we carried out a gene ontology analysis from our RNA sequencing dataset and found that the genetic family representing ‘phosphoric ester hydrolase activity’ was significantly over‐represented in established hypertension (Davis *et al*. 2018; Bardsley *et al*. 2018 *a*), supporting preclinical reports and several clinical studies (Katz *et al*. 2000; Bender, 2006; Nagendran *et al*. 2007; Zaccolo & Movsesian, 2007; Lee *et al*. 2015; Maass *et al*. 2015; Zoccarato *et al*. 2015; Boda *et al*. 2016; Vettel *et al*. 2017; Assenza *et al*. 2018; Baliga *et al*. 2018; Bardsley *et al*. 2018 *a*). It was observed that over 30 genes linked to the PDE superfamily are differentially expressed in the SHR stellate ganglia and that many of these mapped to regulators of PDE activity (Bardsley *et al*. 2018 *a*; Fig. 4 *B*), adding a further layer of complexity to the systems involved in cN control. Moreover, changes in transcripts do not necessarily lead to changes in protein levels. For example, RNA sequencing data revealed a decrease in *Pde2a* expression (Bardsley *et al*. 2018 *a*), whereas PDE2A activity and protein levels are reportedly raised in SHR and human stellates (Li *et al*. 2015; Liu *et al*. 2018). Furthermore, over‐expression of PDE2A in neuronal stellates recapitulates the Ca^2+^ phenotype and enhanced sympathetic response seen in disease (Li *et al*. 2015), illustrating complex interactions that may be related to microdomain signalling of various isoforms of PDE2A (Zhao *et al*. 2016).

### Phosphodiesterases in the myocardium

Within the cardiac sympathetic axis, intrinsic electrical pacemaker activity arising from the sinoatrial node (SAN) dictates resting heart rate, which is increased by sympathetic noradrenaline and activation of myocardial Gα_s_‐coupled β‐adrenergic receptors (βARs). Elevation in myocardial cAMP–PKA activity regulates a large number of phospho‐sensitive processes (Yaniv *et al*. 2015; Behar *et al*. 2016) and in particular plays a key role in elevating intracellular Ca^2+^ via phosphorylation of the L‐type Ca^2+^ channel (Ca_v_1.2, Ca_v_1.3) (Zhao *et al*. 2016, 2017; Hua *et al*. 2012) as well as phospholamban, which increases Ca^2+^ reuptake by the sarcoplasmic reticulum (SR), facilitating rapid repolarisation (Simmerman & Jones, 1998; Mattiazzi & Kranias, 2014; Akaike *et al*. 2017). Conversely, mediators that elevate cGMP–PKG, such as NO coupled to sGCs or activation of membrane‐bound particulate guanylyl cyclase (pGC) receptors (e.g. ANP, BNP), oppose the actions of cAMP–PKA, thus limiting intracellular Ca^2+^. Sustained elevations in cAMP–PKA activity (Sprenger *et al*. 2015) and/or reductions in cardiac NO–cGMP signalling (Heaton *et al*. 2006; Dawson *et al*. 2008; Baliga *et al*. 2018) that elevate [Ca^2+^]_i_ (Leiser & Fleischer, 1996; Mattiazzi & Kranias, 2014; Zhao *et al*. 2016, 2017) are involved in cardiac remodelling and hypertrophy (Sprenger *et al*. 2015; Zoccarato *et al*. 2015), arrhythmia (Kalla *et al*. 2016) and heart failure (Kaye *et al*. 1995; Mehel *et al*. 2013; Florea & Cohn, 2014). In the SHR model, atrial myocytes display a greater cAMP response to βAR stimulation (Heaton *et al*. 2006), and lower basal levels of NO–cGMP (Heaton *et al*. 2006). Gene transfer approaches targeted to the SAN to up‐regulate neuronal nitric oxide synthase (nNOS) or its anchoring protein CAPON (Lu *et al*. 2015) successfully reduce the surface density and activity of L‐type Ca^2+^ currents (Danson *et al*. 2005) and decrease intracellular concentrations of cAMP via the proposed activation of PDE2a (Danson *et al*. 2005), highlighting a novel therapeutic potential for targeting cNs and their effectors within the myocardium directly. The intricacy of cN regulation, the inability to target specific PDE isoforms that reside in precise intracellular compartments, and the high‐level of functional redundancy observed in the PDE superfamily perhaps help to explain the lack of clinical efficacy achieved by selective PDE inhibitors. Computational protein design, protein engineering and the application of targeted vector systems may provide innovative solutions to these problems.

### Neurohormonal and endocrine signalling: effects on presynaptic sympathetic nerves

Impaired neurohormonal regulation plays a critical role in the pathogenesis and progression of cardiovascular diseases (Malpas, 2010). Plasma and tissue levels of noradrenaline (NA), adrenaline (Adr), angiotensin II (AngII), aldosterone and other mediators are significantly altered in hypertension and heart failure and correlate with the severity of disease (Catt *et al*. 1971; Dang *et al*. 1999; Grassi & Esler, 1999; Romero & Reckelhoff, 1999; Rupp & Jäger, 2001; Schiffer *et al*. 2009; Riet *et al*. 2015; Shinohara *et al*. 2015; Najafi *et al*. 2016). Therapeutics aimed at opposing elevated adrenergic and/or antagonising renin–angiotensin–aldosterone signalling are gold‐standard treatment strategies for blood pressure maintenance (van den Meiracker *et al*. 1995; Hansson *et al*. 1999; White *et al*. 2003; Flack *et al*. 2007; Ram, 2010; Nussberger & Bohlender, 2013; Williams *et al*. 2015; Frishman, 2016; Ghazi & Drawz, 2017; Rubattu *et al*. 2018; Wiysonge *et al*. 2018). Nevertheless, their precise mechanisms of action still remain unclear (Nussberger *et al*. 1986; van den Meiracker *et al*. 1995; Nussberger & Bohlender, 2013; Riet *et al*. 2015; Watanabe *et al*. 2017).

**Figure 5:**
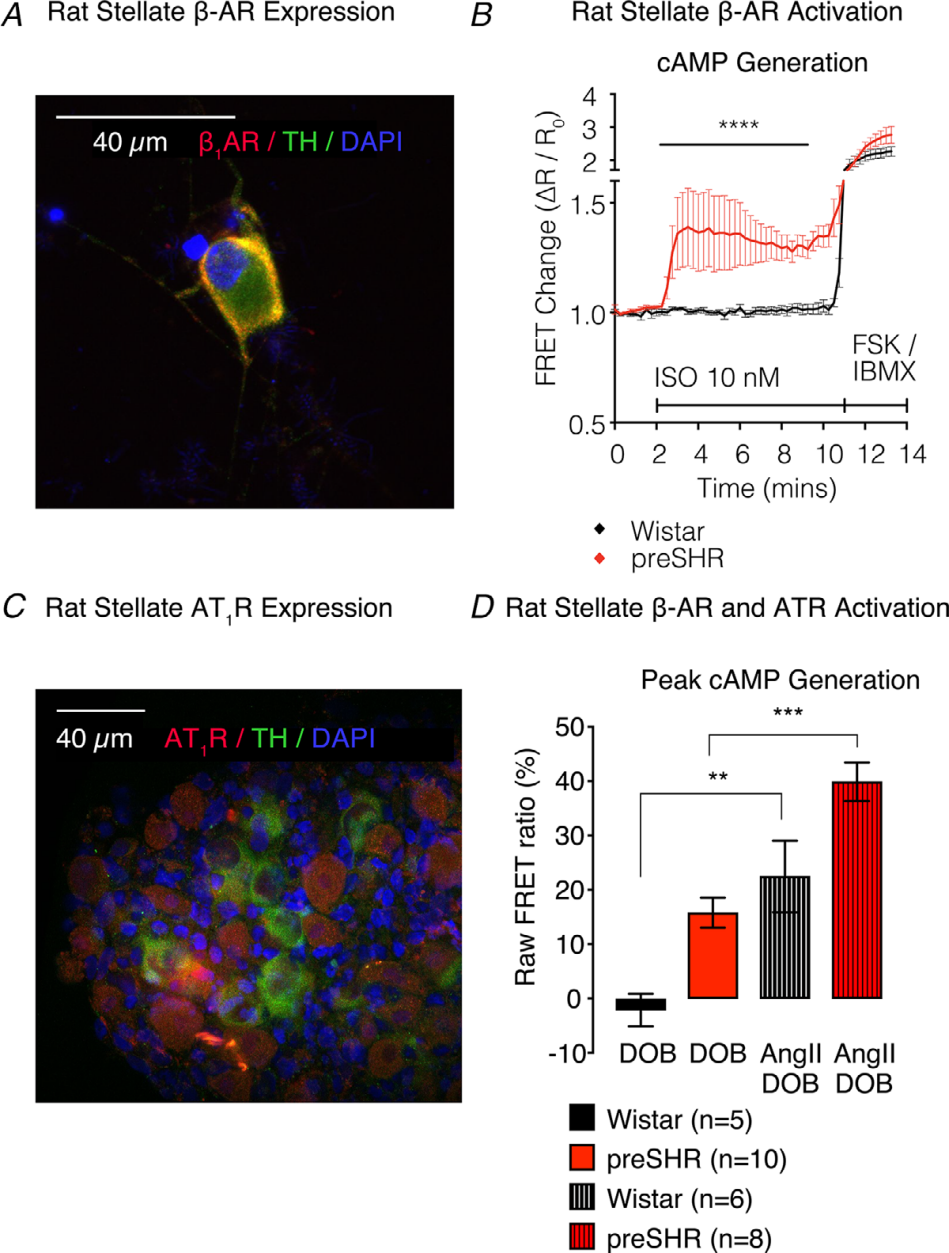
β‐AR signalling is elevated in preSHR neurons *A*, we identified the presence of β‐adrenergic receptors (β‐ARs) on tyrosine hydroxylase (TH) positive cardiac sympathetic neurons. *B*, activation of presynaptic β‐ARs with isoprenaline (10 nM) led to a significantly larger cAMP generation in preSHR (56%; *n* = 12) *vs*. Wistar neurons (7%; *n* = 12; 2‐way ANOVA; *P* < 0.001), which was measured using real‐time cAMP in cells expressing the Epac‐S^H187^ biosensor (*A* and *B* are reproduced from Bardsley *et al*. 2018 *b*). *C*, we also identified the presence of AT_1_Rs on TH positive neurons. *D*, we investigated whether AT1R could elevate B1‐AR‐evoked cAMP. Dobutamine (DOB) alone elevated cAMP in preSHR neurons (reproduced from Bardsley *et al*. 2018 *b*). Moreover, AngII augments DOB‐evoked cAMP generation in Wistar neurons (*n* = 5, 6; *P* = 0.0073) and SHR neurons (*n* = 10, 8; *P* = 0.0005). We also measured a strain‐dependent effect following administration of DOB only (*P* = 0.0015) and in the presence of DOB with AngII (*P* = 0.0283). Data are represented as mean ± SEM.

**Figure 6:**
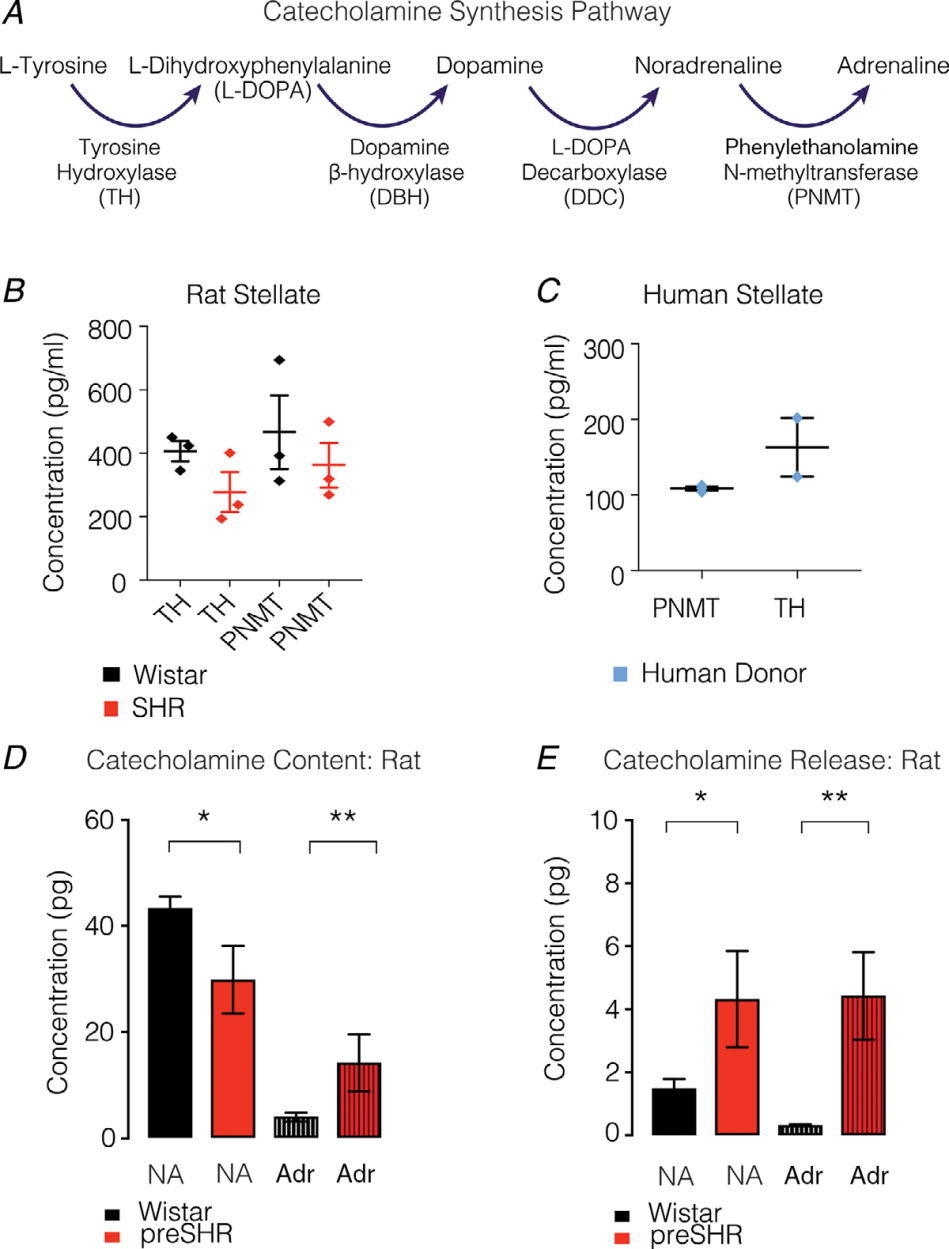
Adrenaline is released from preSHR neurons *A*, the catecholamine synthesis pathway, highlights the role of Phenylethanolamine‐*N*‐methyltransferase (PNMT) in the conversion from noradrenaline (NA) to Adrenaline (Adr). *B* and *C*, tyrosine hydroxylase (TH) and PNMT were measured in adult rat (*B*) and human (*C*) stellate ganglia (reproduced from Bardsley *et al*. 2018 *b*). *D*, using high pressure liquid chromatography with electrochemical detection (HPLC‐EC), we measured significantly higher total NA in Wistar (43.3 ± 2.173 pg; *n* = 8) compared with preSHR neurons (29.82 ± 6.366 pg; *n* = 4; *P* = 0.0294). In the same samples, we also measured a significantly greater total content of Adr in preSHR (14.14 ± 5.399 pg) compared with that measured in Wistar ganglia (3.937 ± 0.820 pg, *P* = 0.0019). *E*, electrical field stimulation of whole rat stellate ganglia led to the release of NA that was significantly higher in samples obtained from preSHR (4.32 ± 1.523 pg) *vs*. Wistar ganglia (1.477 ± 0.316 pg; *P* = 0.0396). The concentrations of neurally mediated Adr release were also significantly higher in preSHR (4.424 ± 1.391 pg, *n* = 4) compared with Wistar stellates (0.3201 ± 0.0325 pg; *n* = 8; *P* = 0.0028) (figure reproduced from Bardsley *et al*. 2018 *b*).

NA transmission plays a dominant role in vascular constriction and cardiac output (Herring & Paterson, 2018), whereas sustained elevations are involved in hypertension (Shanks *et al*. 2013 *b*), arrhythmia (Meredith *et al*. 1991) and heart failure (Kaye *et al*. 1995; Florea & Cohn, 2014). In the 1980s, it was demonstrated that the activation of presynaptic β‐ARs facilitates transmission within several peripheral ganglia (Lokhandwala & Eikenburg, 1983; Majewski, 1983; Misu & Kubo, 1986; Nedergaard & Abrahamsen, 1990; Apparsundaram & Eikenburg, 1995), yet little is known about the physiological or pathophysiological relevance of these receptors in hypertension. Recently, we demonstrated that activation of sympathetic stellate presynaptic β‐AR receptors leads to cAMP–PKA activation that is significantly elevated in the prehypertensive SHR and is predominantly β_2_‐AR mediated (Bardsley *et al*. 2018 *b*) (Fig. 5). This increase in cAMP–PKA signalling augments high K^+^‐evoked Ca^2+^ liberation in neurons from prehypertensive rats, reflecting ion channel involvement (Bardsley *et al*. 2018 *b*). These findings suggest a feed‐forward potentiating mechanism exists for catecholaminergic regulation of cardiac sympathetic transmission, which exacerbates the cAMP/cGMP imbalance in disease (Fig. 6). To give these observations contextual relevance, we also confirmed the presence of β‐ARs in human stellate ganglia, highlighting an alternative site of action for the efficacy achieved with sustained clinical β‐blocker therapy (Ram, 2010; Frishman, 2016; Wiysonge *et al*. 2018).

The renin–angiotensin system (RAS) is critically involved in blood pressure regulation and fluid volume homeostasis (Hall, 1986; Herring & Paterson, 2018) and alterations in RAS signalling are strongly associated with the aetiology of cardiovascular disease (Dang *et al*. 1999; Weir & Dzau, 1999; Rupp & Jäger, 2001; Crowley *et al*. 2006; Riet *et al*. 2015). AngII is a bioactive product of RAS that is synthesised through sequential cleavage of angiotensinogen and angiotensin I by the enzymes renin and angiotensin converting enzyme (ACE) (Weir & Dzau, 1999). Classically, AngII synthesis was thought to predominantly result from the activity of renal‐derived renin, but emerging evidence has highlighted a critical role for ‘intracrine’ or intracellular RAS synthesis (Re & Bryan, 1984; Re, 2003) within several organ and tissue sites including the brain, heart and vasculature (Phillips *et al*. 1993). AngII and RAS peptide reactivity within the brain is primarily observed in areas involved in sympathetic outflow and blood pressure control, including the paraventricular nucleus of the hypothalamus (Li *et al*. 2012; Biancardi *et al*. 2014) nucleus tractus solitarius (Li *et al*. 2012; Shan *et al*. 2013; Biancardi *et al*. 2014), rostroventral lateral medulla (Li *et al*. 2012; Biancardi *et al*. 2014) and subfornical organ (Hendel & Collister, 2005; Cao *et al*. 2012; Li *et al*. 2012), where the effects of AngII are primarily transduced via activation its cognate G_q_‐coupled receptor AT_1_R (Sakai *et al*. 2004; Tan *et al*. 2004; Zhu *et al*. 2004; Sakai & Sigmund, 2005; Wang *et al*. 2012; Shan *et al*. 2013; Biancardi *et al*. 2014; Young & Davisson, 2015).

Evidence suggests that AngII signalling is enhanced in the CNS in hypertension (Chai *et al*. 1993; Gironacci *et al*. 2004; Schiffer *et al*. 2009; Young & Davisson, 2015; Santos *et al*. 2018), heart failure (Wang *et al*. 2012) and post‐myocardial infarction (Tan *et al*. 2004). AngII also has a direct stimulatory effect on peripheral sympathetic neurons themselves (Cox *et al*. 2000; DiBona, 2000; Ma *et al*. 2001; Fernandez *et al*. 2003; Talaia *et al*. 2006; Wang *et al*. 2012; Berg, 2013). Critically, mice lacking the AngII receptor AT_1_R within catecholaminergic neurons develop fewer pathological effects following chronic AngII infusions. This includes attenuated sympathetic activation, reduced hypertensive responses and amelioration of ventricular hypertrophy (Jancovski *et al*. 2013). Collectively, this demonstrates the potential importance of neuronal AngII–AT_1_R activation in the aetiology of sympathetic overactivity and neurogenic hypertension.

The close relationship between elevated AngII and sympathetic overactivity in cardiovascular disease is intriguing (Hilgers *et al*. 1993; Cox *et al*. 2000; Goldsmith, 2004; Berg, 2013) and has raised questions surrounding membrane level receptor–receptor interactions and cross‐talk between AngII and adrenergic signalling cascades (Grant & McGrath, 1988; Barki‐Harrington *et al*. 2003; Tilley, 2011; Saulière *et al*. 2012; Bellot *et al*. 2015; Liu *et al*. 2017; Tóth *et al*. 2018). Specifically, AT_1_R‐α2c adrenergic receptor (AT_1_R‐α_2c_‐AR) heterodimers have been observed, where activation by NA promotes atypical enhanced cAMP–PKA signalling by converting an α_2c_‐AR autoinhibitory signal to excitatory positive feedback signalling (Bellot *et al*. 2015). Moreover, activation of the AT_1_Rα_2c_‐AR heterodimer facilitates NA hypersecretion and sympathetic overactivity in sympathetic neurons *in vivo* (Bellot *et al*. 2015). Heterodimer formation has also been found to occur between AT_1_R‐β_2_‐AR (Barki‐Harrington *et al*. 2003; Tóth *et al*. 2017), which enhances the membrane stability of β_2_‐AR and prolongs cAMP signalling. These results support our observations that AngII augments presynaptic β‐AR‐evoked cAMP (Fig. 5) and suggests a potential synergistic role for NA–AngII‐mediated effects in provoking sympathetic overactivity in hypertension and cardiovascular pathophysiology (Barki‐Harrington *et al*. 2003; Lourdes González‐Hernández *et al*. 2010; Christensen *et al*. 2011; Berg, 2013; Bellot *et al*. 2015; Liu *et al*. 2017; Tóth *et al*. 2018).

### Alterations in cardiac sympathetic transmitter release

Two simultaneous observations led to the concept of Adr as a pathological entity in the progression of hypertension. First, it was observed that Adr infusions underpin sustained increases in blood pressure post‐infusion (Majewski *et al*. 1981; Brown & Macquin, 1982; Brown & Dollery, 1984); and secondly, that plasma Adr is elevated in hypertensive patients (Franco‐Morselli *et al*. 1977; Brown & Macquin, 1981). Brown & Macquin (1981) proposed the ‘adrenaline hypothesis’ of essential hypertension (Brown & Dollery, 1984), which highlights a dominant role for Adr in facilitating NA release through actions at presynaptic β‐ARs (Abboud *et al*. 1964; Floras *et al*. 1988, 1990). The source of Adr, however, was not fully resolved with reports suggesting chronic neuronal uptake and enhanced release of circulating Adr derived from the adrenals as the primary site (Brown & Macquin, 1981; Majewski, 1983; Horikoshi *et al*. 1985; Blankestijn *et al*. 1988; Misu *et al*. 1988; Floras, 1992; Gudmundsdottir *et al*. 2008). Evidence has pointed to the possible synthesis of Adr in sympathetic nerves in patients with hypertension and stress disorder (Esler *et al*. 2008), but the *in situ* synthesis of Adr and a role for cardiac sympathetic Adr in the aetiology of hypertension are far from well‐established.

Our RNA sequencing dataset provided a comprehensive profile of neurotransmitters and their respective synthesising enzymes in rat stellate ganglia (Bardsley *et al*. 2018 *a*). Alongside the presence of classical transmitters and sympathetic markers, we also observed the transcript encoding phenylethanolamine *N*‐methyltransferase (PNMT), the enzyme involved in the conversion of NA to Adr (Bardsley *et al*. 2018 *a*). Protein concentrations of PNMT were detectable in rat and human stellate ganglia. To ascertain whether the presence of PNMT results in physiological concentrations and release of Adr, we electrically stimulated stellate ganglia from normotensive and hypertensive rats. Levels of both NA and Adr were elevated in the perfusate collected from prehypertensive SHR ganglia, whereas only NA could be detected in perfusate from healthy rat ganglia, and Adr was not observed (Fig. 6; Bardsley *et al*. 2018 *b*). In support of this observation, a 20‐year follow‐up of the Oslo study on normotensive, prehypertensive and male patients with established hypertension has identified arterial Adr as an independent predictor of blood pressure elevation (Gudmundsdottir *et al*. 2008), re‐raising the question of the importance of Adr in the pathophysiology of hypertension (Rumantir *et al*. 2000 *a*). It is now evident that Adr synthesis occurs directly within cardiac sympathetic nerves in diseases associated with sympathetic overactivity (Esler *et al*. 2008), and that this neurotransmitter switching takes place before elevations in arterial blood pressure are observed (Bardsley *et al*. 2018 *b*). In addition to the observed elevation in β‐AR‐mediated cAMP–PKA–Ca^2+^ signalling in prehypertensive rat stellate ganglia, these data support the notion of a causal role for Adr in the pathophysiology of neurogenic hypertension.

### Targeting sympathetic overactivity: where are we now?

Hypertension is central in determining cardiovascular risk and is a strong predictive indicator of morbidity and mortality; however, there still remains an unmet clinical need for disease‐modifying and prophylactic interventions. Cardiac sympathetic hyperactivity is a key feature of human hypertension that is also seen in animal models of cardiovascular disease (Esler, 2010; Larsen *et al*. 2016 *a*), yet interventions that target this sympathetic phenotype are problematic to develop, due to the anatomical location of the cardiac sympathetic ganglia (Kwon *et al*. 2018) and the challenge in unravelling the underlying pathophysiological mechanisms. Surgical techniques such as sympathectomy *per se*, provide symptomatic relief and lead to fewer cardiovascular co‐morbidities in hypertension (Morrissey *et al*. 1953) and reduce the incidence of ventricular arrhythmia (Ajijola *et al*. 2014; Irie *et al*. 2017), yet these techniques are not without risk (Ajijola *et al*. 2014). Current pharmacological approaches including β‐blockers and AngII inhibitors are mainstay therapeutic strategies for early hypertension and many other cardiovascular diseases associated with dysautonomia (Wiysonge *et al*. 2017). However, their efficacy may also be explained via reductions in peripheral sympatho‐transmission. Approaches that aim to modulate sympathetic overactivity may have both a therapeutic and a physiological advantage over surgical techniques. Optimal neuromodulation of sympathetic tone will counteract hypertension‐induced cardiovascular damage whilst retaining a level of sympathetic reserve that will still enable cardiac performance during physical exertion. Gene transfer therapies that modulate cyclic nucleotide activity have had some success in improving neuronal activity, and a new era of genetic and protein modification techniques might be predicted to underpin the primary areas of advancement in this field. Moreover, the application of bioinformatics and the integration of machine‐learning techniques with primary research may provide novel approaches for assisting diagnoses and prediction (LaFreniere *et al*. 2016; Kublanov *et al*. 2017; Savage, 2017; Poplin *et al*. 2018) as well as providing clarity regarding the complex interactions between pathways and their associated cellular and molecular processes (Cunningham, 2017; Wang *et al*. 2017; Xie *et al*. 2017; Cholley *et al*. 2018; Costello & Martin, 2018; Pavillon *et al*. 2018), as a way to facilitate precise therapeutic targeting.

## Conclusion

Sympathetic overactivity is a well‐established contributor to hypertension and CVD. Increased intracellular Ca^2+^ augments neurotransmission early in disease before increases in blood pressure develop. This Ca^2+^ phenotype is underpinned by an impaired cAMP/cGMP balance that is weighted in favour of cAMP–PKA‐dependent activity. Evidence suggests that this alteration in cN signalling results from changes in presynaptic receptor expression and signalling pathways, as well as critical changes in PDE activity. Pharmacological, surgical and genetic techniques aimed at reducing sympathetic tone or raising vagal transmission have had reasonable levels of success reducing hypertension and improving cardiac function (Morrissey *et al*. 1953; Heaton *et al*. 2007; Sabbah *et al*. 2011; Rathi *et al*. 2013; Ajijola *et al*. 2014; Sverrisdottir *et al*. 2014; Shivkumar *et al*. 2016; Irie *et al*. 2017); nevertheless, no prophylactic strategies have yet successfully entered the clinical arena, emphasising a critical need for translational advancements in this field.

## Additional information

### Competing interests

None of the authors have any conflicts of interests.

### Author contributions

Both authors have read and approved the final version of this manuscript and agree to be accountable for all aspects of the work in ensuring that questions related to the accuracy or integrity of any part of the work are appropriately investigated and resolved. All persons designated as authors qualify for authorship, and all those who qualify for authorship are listed.

### Funding information

This work was funded by the Wellcome Trust OXION (105409/Z/14/Z) and British Heart Foundation (RG/17/14/33085).
